# Crystal structure of a palladium(II) complex containing the wide bite-angle bis­(selenium) ligand, *cis*-[(^*t*^BuNH)(Se)P(μ-N^*t*^Bu)_2_P(Se)(NH^*t*^Bu)]

**DOI:** 10.1107/S2056989018000841

**Published:** 2018-01-19

**Authors:** Austin Bonnette, Joel T. Mague, Perumalreddy Chandrasekaran

**Affiliations:** a Department of Chemistry and Biochemistry, Lamar University, Beaumont, TX 77710, USA; b Department of Chemistry, Tulane University, 6400 Freret Street, New Orleans, LA, 70118, USA

**Keywords:** crystal structure, selenium ligand, palladium(II) complex, P-N compounds, cyclo­diphosphaza­nes, bite-angle

## Abstract

A palladium(II) complex {systematic name: dichlorido[1,3-di-*tert*-butyl-2,4-bis(*tert*-butylamino)-1,3,2λ^5^,4λ^5^-diazadiphosphetidine-2,4-diselone-κ^2^ *Se*,*Se*′]pal­ladium(II)}, *cis*-[PdCl_2_{**I**}], (**II**), containing a bis­(selenium) ligand based on cyclo­diphosph(V)azane, *cis*-[(^*t*^BuNH)(Se)P(μ-N^*t*^Bu)_2_P(Se)(NH^*t*^Bu)], (**I**), has been synthesized and structurally characterized. The crystal structure of complex **II** reveals that the ligand chelates through selenium donors with the natural bite-angle of 110.54 (1)° and a Pd—Se bond distance of 2.444 (1) Å. The coordination around Pd^II^ shows a slightly distorted square-planar geometry, as indicated by the angle between the [PdCl_2_] and [PdSe_2_] planes of 5.92 (3)°. In the crystal, the mol­ecules are inter­linked through weak N—H⋯Cl and C—H⋯Cl hydrogen-bonding inter­actions.

## Abstract 2

A palladium(II) complex containing a bis­(selenium) ligand based on cyclo­diphosph(V)azane, *cis*-[(^*t*^BuNH)(Se)P(μ-N^*t*^Bu)_2_P(Se)(NH^*t*^Bu)] has been synthesized and structurally characterized. The crystal structure revels chelation of ligand through selenium donors with a natural bite-angle of 110.54 (1)°

## Chemical context   

Cyclo­diphosph(III)aza­nes are four-membered P^III^–N ring systems with general formula, *cis*-[*R*P(μ-N^*t*^Bu)_2_P*R*]. The planar nature of the four-membered ring favors a bridging bidentate coordination mode through phospho­rus donors rather than chelation, to afford structurally inter­esting macrocyclic and polymeric complexes (Balakrishna, 2016 ▸). The main-group chemistry of the corresponding P^V^ analogue cyclo­diphosph(V)aza­nes, *cis*-[*R*(*E*)P(μ-N^*t*^Bu)_2_P(*E*)*R*] (*E* = O, S, Se, and Te; *R* = NH^*t*^Bu) and its amide derivatives has been studied extensively by Stahl (2000 ▸) and Briand and co-workers (Briand *et al.*, 2002 ▸). While examples of coordination of cyclo­diphosph(V)aza­nes with transition metal ions are scarce, the sulfur and selenium derivatives are especially inter­esting as they have a special affinity for soft metals and have the potential to form complexes with wide natural bite-angles through chelation (Chivers *et al.*, 2001 ▸). Several late transition-metal complexes containing wide natural bite-angle chelating ligands (*L*—*M*—*L =* 100–134°) have been developed over the years and have shown promising catalytic activity for several reactions (Kamer *et al.*, 2001 ▸). The majority of these wide bite-angle ligands are phospho­rus and/or nitro­gen donor ligands (Motolko *et al.*, 2017 ▸; Czauderna *et al.*, 2015 ▸). Herein we report the synthesis and crystal structure of the palladium(II) complex (**II**) with a wide bite-angle selenium ligand based on cyclo­diphosph(V)azane *cis*-[(^*t*^BuNH)(Se)P(μ-N^*t*^Bu)_2_P(Se)(NH^*t*^Bu)], (**I**).
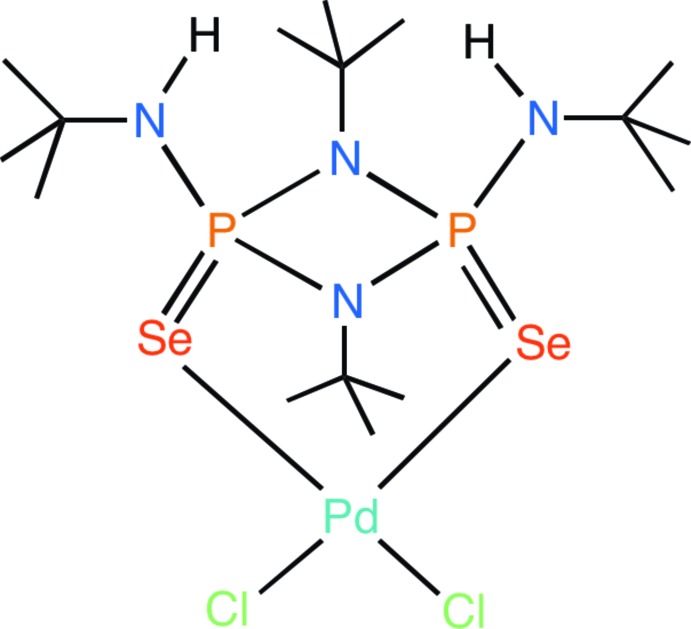


## Structural commentary   

**Figure 1:**
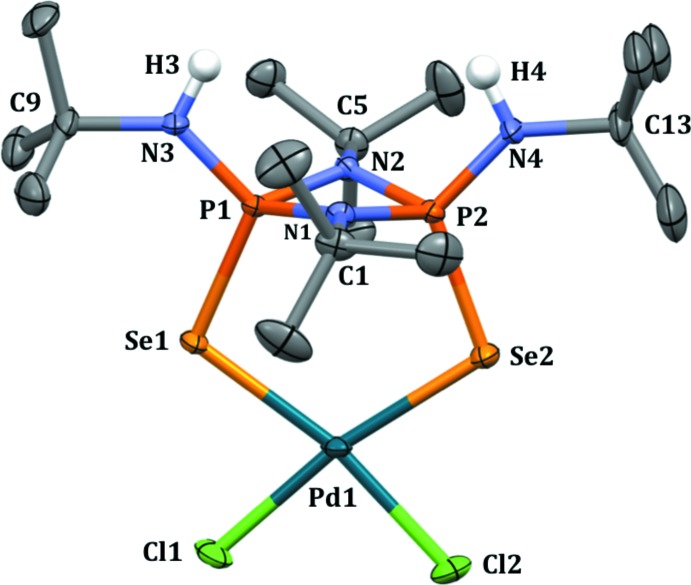
Perspective view of palladium complex **II** with displacement ellipsoids drawn at the 50% probability level. All H atoms have been omitted for clarity except at N3 and N4. Only the major occupancy component of the disordered *t*-butyl group is shown.

A perspective view of the mol­ecular structure of the Pd^II^ complex (**II**) is presented in Fig. 1 ▸. The crystal structure of **II** confirms the chelation of *cis*-[(^*t*^BuNH)(Se)P(μ-N^*t*^Bu)_2_P(Se)(NH^*t*^Bu)] (**I**) through selenium donors to the [PdCl_2_] moiety, with a Se1—Pd1—Se2 natural bite-angle of 110.54 (1)°. The rigid four-membered cyclo­diphosphazane [P(μ-N^*t*^Bu)_2_P] ligand backbone enforces large natural bite-angles. The Se1—Pd1—Cl1 and Se2—Pd1—Cl2 bond angles are 79.27 (2) and 79.36 (2)°, respectively, smaller than the natural square-planar angle, whereas the Cl1—Pd1—Cl2 angle [91.19 (2)°] is closer to the typical value for a square-planar angle. In complex **II**, the exocyclic Se1—P1—N3 and Se2—P2—N4 angles at 114.32 (7) and 117.13 (7)°, respectively, are slightly larger than the corresponding angle in the uncoordin­ated ligand **I** [107.3 (1) and 113.2 (1)°; Chivers *et al.*, 2002 ▸]. In complex **II**, the palladium atom shows a slight tetra­hedral distortion from a square-planar geometry, as indicated by the dihedral angle between the Se1/Pd1/Se2 and Cl1/Pd1/Cl2 planes of 5.92 (3)°. The Pd1—Se1 and Pd1—Se2 bond distances are 2.4458 (3) and 2.4440 (3) Å, respectively, and are in the typical range for Pd^II^ complexes with selenium ligands (Das *et al.*, 2009 ▸). In complex **II**, the P1—Se1 and P2—Se2 bond distances are 2.1543 (6) and 2.1654 (6) Å, respectively; these bonds are slightly elongated compared to the P—Se bond [2.078 (1) Å] in the uncoordinated ligand (**I**). This may be a result of the coordination of Se to the Pd center. The Pd—Cl bond distances [Pd1—Cl1 = 2.3381 (6) and Pd1—Cl2 = 2.3159 (6) Å] are consistent with those reported for Pd^II^ complexes with Se donor ligands (Saleem *et al.*, 2013 ▸). The [P(μ-N^*t*^Bu)_2_P] ring in complex **II** is greatly puckered, as indicated by the angle of 22.61 (2)° between the N1/P1/N2 and N1/P2/N2 planes. The corresponding dihedral angle for the uncoordinated ligand is 3.73 (2)°.

## Supra­molecular features   

**Figure 2:**
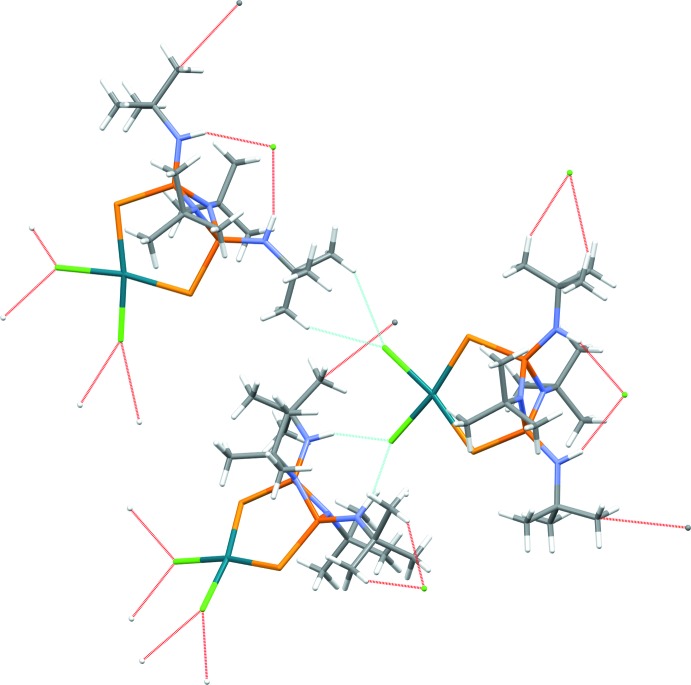
Hydrogen-bonding inter­actions in the crystal lattice.

**Figure 3:**
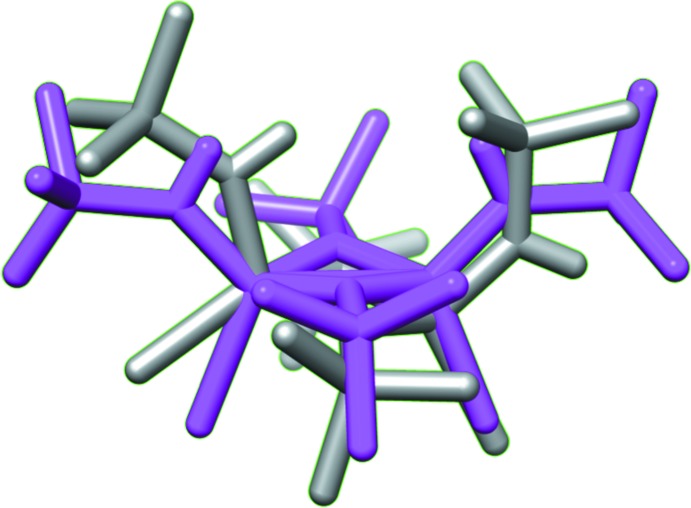
Overlay of the uncoordinated ligand **I** (gray) with the coordinated ligand fragment in complex **II** (purple).

In the crystal, the mol­ecules are connected through weak N—H⋯Cl and C—H⋯Cl hydrogen-bonding inter­actions (Fig. 2 ▸, Table 1 ▸). Inter­estingly, in the solid-state structure **II**, the exocyclic nitro­gen substitutents are arranged in an *endo*, *endo* fashion, whereas in ligand **I** they are arranged in *exo, endo* orientations (Chivers *et al.*, 2002 ▸). An overlay plot of the free ligand mol­ecule **I** with the ligand fragment of **II** is shown in Fig. 3 ▸. This conformational change upon coordination is possibly caused by the formation of inter­molecular hydrogen-bonding inter­actions. A similar conformational change influenced by hydrogen-bonding inter­actions has previously been noted (Chandrasekaran *et al.*, 2011 ▸).

## Synthesis and crystallization   

The ligand *cis*-[(^*t*^BuHN)(Se)P(μ-^*t*^BuN)_2_P(Se)(NH^*t*^Bu)], (**I**), was prepared following a reported procedure (Chivers *et al.*, 2002 ▸).

A di­chloro­methane solution (10 mL) of [Pd(COD)Cl_2_] (100 mg, 0.35 mmol) was added dropwise to a solution of *cis*-[(^*t*^BuHN)(Se)P(μ-^*t*^BuN)_2_P(Se)(NH^*t*^Bu)] (175 mg, 0.35 mmol) in 10 mL of CH_2_Cl_2_ under an N_2_ atmosphere at ambient temperature. The resultant dark-orange solution was stirred for 6 h. The solution was then concentrated to 10 mL, diluted with 10 mL of pentane, and stored at 248 K for a day to afford the analytically pure orange crystalline product. X-ray quality crystals were obtained by slow evaporation from a DMF solution at room temperature. Yield: 76% (206 mg, 0.067 mmol), m.p. 455–457 K.

^1^H NMR (400 MHz, DMSO-*d*_6_): 1.44 (*s*, 18H, ^*t*^Bu), 1.57 (*s*, 18H, ^*t*^Bu), 2.3 (*br s*, 2H, NH). IR (cm^−1^): 3175 (*br w*), 2974 (*w*), 1469 (*w*), 1392 (*w*), 1367 (*m*), 1367 (*m*), 1227 (*m*), 1184 (*s*), 1028 (*s*), 893 (*s*), 837 (*w*), 733 (*m*), 683 (*m*). Absorption spectrum [CH_2_Cl_2_; λ_max_, nm (∊_M_, M^−1^cm^−1^)]: 247 (12068), 294 (15752), 355 (6827). Analysis calculated for C_16_H_38_N_4_P_2_Se_2_PdCl_2_: C, 28.11; H, 5.60; N, 8.19. Found: C, 28.37; H, 6.01; N, 28.74.

## Refinement   

Crystal data, data collection and structure refinement details are summarized in Table 2 ▸. All H atoms attached to carbon were placed in calculated positions (C—H = 0.98 Å), while those attached to nitro­gen were placed in locations derived from a difference-Fourier map and their coordinates adjusted to give N—H = 0.91 Å. All were included as riding contributions with *U*_iso_(H) = 1.2-1.5 times those of the parent atoms. The *t*-butyl group attached to N4 was modeled as rotationally disordered over two sites of approximately equal population. These were refined with restraints so that the geometries of the two components of the disorder are comparable.

## Supplementary Material

Crystal structure: contains datablock(s) global, I. DOI: 10.1107/S2056989018000841/nk2239sup1.cif

Structure factors: contains datablock(s) I. DOI: 10.1107/S2056989018000841/nk2239Isup2.hkl

CCDC reference: 1549758

Additional supporting information:  crystallographic information; 3D view; checkCIF report

## Figures and Tables

**Table 1:** Hydrogen-bond geometry (Å, °)

*D*—H⋯*A*	*D*—H	H⋯*A*	*D*⋯*A*	*D*—H⋯*A*
N4—H4⋯Cl1^i^	0.91	2.45	3.317 (2)	159
N3—H3⋯Cl1^i^	0.91	2.57	3.4160 (19)	155
C16—H16*A*⋯Cl2^ii^	0.98	2.82	3.746 (5)	157
C14*A*—H14*E*⋯Cl2^ii^	0.98	2.82	3.742 (7)	157

Symmetry codes: (i) 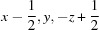; (ii) 
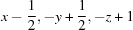
.

**Table 2:** Experimental details

Crystal data	
Chemical formula	[PdCl_2_(C_16_H_38_N_4_P_2_Se_2_)]
*M*_r_	683.66
Crystal system, space group	Orthorhombic, *P* *b* *c* *a*
Temperature (K)	150
*a*, *b*, *c* (Å)	17.3733 (10), 15.7184 (9), 19.5052 (11)
*V* (Å^3^)	5326.5 (5)
*Z*	8
Radiation type	Mo *K*α
μ (mm^−1^)	3.76
Crystal size (mm)	0.18 × 0.13 × 0.12

Data collection	
Diffractometer	Bruker SMART APEX CCD
Absorption correction	Numerical (*SADABS*; Bruker, 2013 ▸)
*T*_min_, *T*_max_	0.44, 0.66
No. of measured, independent and observed [*I* > 2σ(*I*)] reflections	94164, 7112, 6053
*R*_int_	0.053
(sin θ/λ)_max_ (Å^−1^)	0.686

Refinement	
*R*[*F* ^2^ > 2σ(*F* ^2^)], *wR*(*F* ^2^), *S*	0.027, 0.064, 1.03
No. of reflections	7112
No. of parameters	251
No. of restraints	45
H-atom treatment	H-atom parameters constrained
Δρ_max_, Δρ_min_ (e Å^−3^)	1.10, −0.82

Computer programs: *APEX2* and *SAINT* (Bruker, 2013 ▸), *SHELXT* (Sheldrick, 2015*a* ▸), *SHELXL2014* (Sheldrick, 2015*b* ▸), *Mercury* (Macrae *et al.*, 2006 ▸) and *SHELXTL* (Sheldrick, 2008 ▸).

## supplementary crystallographic information

### Crystal data

**Table:**

[PdCl_2_(C_16_H_38_N_4_P_2_Se_2_)]	*D*_x_ = 1.705 Mg m^−^^3^
*M**_r_* = 683.66	Mo *K*α radiation, λ = 0.71073 Å
Orthorhombic, *P**b**c**a*	Cell parameters from 9629 reflections
*a* = 17.3733 (10) Å	θ = 2.4–29.1°
*b* = 15.7184 (9) Å	µ = 3.76 mm^−^^1^
*c* = 19.5052 (11) Å	*T* = 150 K
*V* = 5326.5 (5) Å^3^	Block, orange
*Z* = 8	0.18 × 0.13 × 0.12 mm
*F*(000) = 2720	

### Data collection

**Table:**

Bruker SMART APEX CCD diffractometer	7112 independent reflections
Radiation source: fine-focus sealed tube	6053 reflections with *I* > 2σ(*I*)
Graphite monochromator	*R*_int_ = 0.053
Detector resolution: 8.3660 pixels mm^-1^	θ_max_ = 29.2°, θ_min_ = 2.0°
φ and ω scans	*h* = −23→23
Absorption correction: numerical (*SADABS*; Bruker, 2013)	*k* = −21→21
*T*_min_ = 0.44, *T*_max_ = 0.66	*l* = −26→26
94164 measured reflections	

### Refinement

**Table:**

Refinement on *F*^2^	Primary atom site location: structure-invariant direct methods
Least-squares matrix: full	Secondary atom site location: difference Fourier map
*R*[*F*^2^ > 2σ(*F*^2^)] = 0.027	Hydrogen site location: mixed
*wR*(*F*^2^) = 0.064	H-atom parameters constrained
*S* = 1.03	*w* = 1/[σ^2^(*F*_o_^2^) + (0.0283*P*)^2^ + 6.7911*P*] where *P* = (*F*_o_^2^ + 2*F*_c_^2^)/3
7112 reflections	(Δ/σ)_max_ = 0.002
251 parameters	Δρ_max_ = 1.10 e Å^−^^3^
45 restraints	Δρ_min_ = −0.82 e Å^−^^3^

### Special details

**Table:**

Experimental. The diffraction data were obtained from 3 sets of 400 frames, each of width 0.5° in ω, collected at φ = 0.00, 90.00 and 180.00° and 2 sets of 800 frames, each of width 0.45° in φ, collected at ω = –30.00 and 210.00°. The scan time was 10 sec/frame.
Geometry. All esds (except the esd in the dihedral angle between two l.s. planes) are estimated using the full covariance matrix. The cell esds are taken into account individually in the estimation of esds in distances, angles and torsion angles; correlations between esds in cell parameters are only used when they are defined by crystal symmetry. An approximate (isotropic) treatment of cell esds is used for estimating esds involving l.s. planes.
Refinement. Refinement of F^2^ against ALL reflections. The weighted R-factor wR and goodness of fit S are based on F^2^, conventional R-factors R are based on F, with F set to zero for negative F^2^. The threshold expression of F^2^ > 2sigma(F^2^) is used only for calculating R-factors(gt) etc. and is not relevant to the choice of reflections for refinement. R-factors based on F^2^ are statistically about twice as large as those based on F, and R- factors based on ALL data will be even larger. H-atoms attached to carbon were placed in calculated positions (C—H = 0.98 Å) while those attached to nitrogen were placed in locations derived from a difference map and their coordinates adjusted to give N—H = 0.91 Å. All were included as riding contributions with isotropic displacement parameters 1.2 - 1.5 times those of the attached atoms. The *t*-butyl group attached to N4 is rotationally disordered over two sites of approximately equal population. These were refined with restraints that the geometries of the two components of the disorder be comparable.

### Fractional atomic coordinates and isotropic or equivalent isotropic displacement parameters (Å^2^)

**Table:**

	*x*	*y*	*z*	*U*_iso_*/*U*_eq_	Occ. (<1)
Pd1	0.82807 (2)	0.37109 (2)	0.32897 (2)	0.01569 (5)	
Se1	0.79075 (2)	0.43142 (2)	0.21827 (2)	0.01764 (6)	
Se2	0.71420 (2)	0.33024 (2)	0.39476 (2)	0.02033 (6)	
Cl1	0.94805 (3)	0.41297 (5)	0.28642 (3)	0.02943 (14)	
Cl2	0.88507 (4)	0.31003 (5)	0.42436 (3)	0.03151 (15)	
P1	0.66761 (3)	0.41544 (4)	0.21683 (3)	0.01258 (10)	
P2	0.61982 (3)	0.35870 (4)	0.32597 (3)	0.01408 (11)	
N1	0.63567 (10)	0.32168 (11)	0.24642 (9)	0.0142 (3)	
N2	0.62529 (10)	0.45475 (11)	0.28785 (9)	0.0138 (3)	
N3	0.62727 (10)	0.44265 (13)	0.14563 (9)	0.0180 (4)	
H3	0.575137	0.441740	0.150051	0.022*	
N4	0.53418 (11)	0.33921 (13)	0.35323 (10)	0.0203 (4)	
H4	0.498851	0.355468	0.321389	0.024*	
C1	0.64163 (14)	0.23290 (15)	0.21846 (13)	0.0224 (5)	
C2	0.72478 (16)	0.21282 (19)	0.20073 (19)	0.0418 (8)	
H2A	0.741606	0.249383	0.162896	0.063*	
H2B	0.729027	0.153044	0.186953	0.063*	
H2C	0.757368	0.223065	0.240900	0.063*	
C3	0.5901 (2)	0.22733 (19)	0.15545 (16)	0.0417 (7)	
H3A	0.537340	0.242791	0.168043	0.063*	
H3B	0.590870	0.169072	0.137562	0.063*	
H3C	0.609019	0.266535	0.120197	0.063*	
C4	0.61299 (18)	0.17126 (17)	0.27283 (16)	0.0358 (6)	
H4A	0.644908	0.176515	0.314045	0.054*	
H4B	0.616222	0.112953	0.255264	0.054*	
H4C	0.559394	0.184560	0.284242	0.054*	
C5	0.62346 (14)	0.54289 (15)	0.31730 (12)	0.0201 (5)	
C6	0.55892 (19)	0.54603 (18)	0.36997 (16)	0.0422 (8)	
H6A	0.510622	0.527885	0.348517	0.063*	
H6B	0.553355	0.604315	0.387109	0.063*	
H6C	0.571202	0.507919	0.408193	0.063*	
C7	0.69939 (18)	0.56807 (18)	0.35043 (16)	0.0361 (7)	
H7A	0.710827	0.529084	0.388296	0.054*	
H7B	0.695567	0.626300	0.368064	0.054*	
H7C	0.740675	0.565045	0.316289	0.054*	
C8	0.60370 (16)	0.60309 (16)	0.25870 (14)	0.0289 (5)	
H8A	0.644157	0.600390	0.223758	0.043*	
H8B	0.599801	0.661335	0.276281	0.043*	
H8C	0.554417	0.586272	0.238353	0.043*	
C9	0.65722 (14)	0.46705 (17)	0.07581 (11)	0.0235 (5)	
C10	0.70489 (17)	0.3951 (2)	0.04504 (14)	0.0360 (6)	
H10A	0.750172	0.384783	0.073815	0.054*	
H10B	0.721499	0.411007	−0.001237	0.054*	
H10C	0.673625	0.343287	0.042754	0.054*	
C11	0.58469 (16)	0.4814 (2)	0.03314 (13)	0.0355 (7)	
H11A	0.553537	0.429424	0.033088	0.053*	
H11B	0.599201	0.495780	−0.014006	0.053*	
H11C	0.554791	0.528195	0.052961	0.053*	
C12	0.70387 (17)	0.54828 (19)	0.07905 (14)	0.0338 (6)	
H12A	0.672869	0.593370	0.100063	0.051*	
H12B	0.718703	0.565358	0.032569	0.051*	
H12C	0.750228	0.538698	0.106606	0.051*	
C13	0.49714 (15)	0.30323 (18)	0.41632 (12)	0.0295 (6)	
C14	0.4321 (4)	0.2471 (5)	0.3901 (3)	0.0556 (8)	0.548 (3)
H14A	0.401481	0.278775	0.356477	0.083*	0.548 (3)
H14B	0.453769	0.196251	0.368310	0.083*	0.548 (3)
H14C	0.399206	0.230116	0.428518	0.083*	0.548 (3)
C15	0.4492 (4)	0.3798 (4)	0.4498 (3)	0.0556 (8)	0.548 (3)
H15A	0.414712	0.404302	0.415257	0.083*	0.548 (3)
H15B	0.418821	0.358301	0.488360	0.083*	0.548 (3)
H15C	0.484838	0.423715	0.466105	0.083*	0.548 (3)
C16	0.5472 (3)	0.2673 (5)	0.4654 (3)	0.0556 (8)	0.548 (3)
H16A	0.517051	0.245880	0.504238	0.083*	0.548 (3)
H16B	0.575834	0.220258	0.444540	0.083*	0.548 (3)
H16C	0.583308	0.310757	0.481535	0.083*	0.548 (3)
C14A	0.4149 (4)	0.3018 (6)	0.4084 (4)	0.0556 (8)	0.452 (3)
H14D	0.401634	0.273183	0.365383	0.083*	0.452 (3)
H14E	0.391776	0.271054	0.446940	0.083*	0.452 (3)
H14F	0.395302	0.360261	0.407521	0.083*	0.452 (3)
C15A	0.5291 (4)	0.3444 (5)	0.4791 (3)	0.0556 (8)	0.452 (3)
H15D	0.585444	0.341047	0.478162	0.083*	0.452 (3)
H15E	0.513235	0.404196	0.480458	0.083*	0.452 (3)
H15F	0.509709	0.314989	0.519877	0.083*	0.452 (3)
C16A	0.5256 (4)	0.2064 (4)	0.4239 (4)	0.0556 (8)	0.452 (3)
H16D	0.581653	0.205263	0.429416	0.083*	0.452 (3)
H16E	0.501260	0.180603	0.464190	0.083*	0.452 (3)
H16F	0.511218	0.174341	0.382781	0.083*	0.452 (3)

### Atomic displacement parameters (Å^2^)

**Table:**

	*U*^11^	*U*^22^	*U*^33^	*U*^12^	*U*^13^	*U*^23^
Pd1	0.00934 (8)	0.02154 (9)	0.01619 (8)	0.00250 (6)	−0.00299 (6)	−0.00155 (6)
Se1	0.00884 (10)	0.02754 (13)	0.01654 (10)	−0.00158 (8)	−0.00016 (7)	0.00226 (8)
Se2	0.01424 (11)	0.02861 (13)	0.01812 (11)	−0.00049 (9)	−0.00335 (8)	0.00731 (9)
Cl1	0.0094 (2)	0.0534 (4)	0.0255 (3)	−0.0011 (2)	−0.0001 (2)	−0.0038 (3)
Cl2	0.0230 (3)	0.0455 (4)	0.0260 (3)	0.0080 (3)	−0.0106 (2)	0.0073 (3)
P1	0.0090 (2)	0.0153 (3)	0.0134 (2)	0.00044 (19)	−0.00020 (18)	0.00051 (19)
P2	0.0099 (2)	0.0170 (3)	0.0154 (2)	−0.0007 (2)	−0.00030 (19)	0.00305 (19)
N1	0.0138 (8)	0.0129 (9)	0.0159 (8)	0.0001 (7)	0.0002 (7)	−0.0006 (6)
N2	0.0127 (8)	0.0136 (9)	0.0150 (8)	0.0007 (7)	0.0022 (6)	0.0009 (6)
N3	0.0101 (8)	0.0276 (11)	0.0161 (8)	0.0001 (7)	−0.0021 (7)	0.0044 (7)
N4	0.0115 (9)	0.0320 (11)	0.0173 (9)	−0.0032 (8)	0.0008 (7)	0.0094 (8)
C1	0.0206 (11)	0.0143 (11)	0.0323 (13)	0.0007 (9)	−0.0015 (9)	−0.0062 (9)
C2	0.0291 (15)	0.0266 (15)	0.070 (2)	0.0043 (12)	0.0118 (14)	−0.0188 (14)
C3	0.054 (2)	0.0257 (15)	0.0453 (17)	−0.0056 (14)	−0.0199 (15)	−0.0098 (12)
C4	0.0391 (16)	0.0162 (13)	0.0520 (18)	−0.0035 (11)	−0.0010 (13)	0.0026 (11)
C5	0.0247 (12)	0.0152 (11)	0.0204 (11)	0.0031 (9)	0.0029 (9)	−0.0033 (8)
C6	0.055 (2)	0.0264 (14)	0.0454 (17)	0.0077 (14)	0.0282 (15)	−0.0056 (12)
C7	0.0407 (16)	0.0257 (14)	0.0420 (16)	0.0014 (12)	−0.0149 (13)	−0.0123 (12)
C8	0.0333 (14)	0.0178 (12)	0.0357 (14)	0.0025 (11)	−0.0008 (11)	0.0037 (10)
C9	0.0217 (11)	0.0364 (14)	0.0125 (10)	−0.0039 (10)	−0.0019 (8)	0.0050 (9)
C10	0.0337 (15)	0.0534 (19)	0.0207 (12)	0.0019 (13)	0.0058 (11)	−0.0034 (12)
C11	0.0314 (14)	0.0517 (18)	0.0235 (12)	−0.0045 (13)	−0.0128 (11)	0.0115 (12)
C12	0.0344 (15)	0.0417 (16)	0.0251 (13)	−0.0143 (13)	−0.0037 (11)	0.0102 (11)
C13	0.0284 (13)	0.0402 (15)	0.0197 (11)	−0.0126 (12)	0.0065 (10)	0.0086 (10)
C14	0.0535 (17)	0.076 (2)	0.0375 (15)	−0.0114 (17)	0.0209 (13)	0.0186 (14)
C15	0.0535 (17)	0.076 (2)	0.0375 (15)	−0.0114 (17)	0.0209 (13)	0.0186 (14)
C16	0.0535 (17)	0.076 (2)	0.0375 (15)	−0.0114 (17)	0.0209 (13)	0.0186 (14)
C14A	0.0535 (17)	0.076 (2)	0.0375 (15)	−0.0114 (17)	0.0209 (13)	0.0186 (14)
C15A	0.0535 (17)	0.076 (2)	0.0375 (15)	−0.0114 (17)	0.0209 (13)	0.0186 (14)
C16A	0.0535 (17)	0.076 (2)	0.0375 (15)	−0.0114 (17)	0.0209 (13)	0.0186 (14)

### Geometric parameters (Å, º)

**Table:**

Pd1—Cl2	2.3159 (6)	C8—H8A	0.9800
Pd1—Cl1	2.3381 (6)	C8—H8B	0.9800
Pd1—Se2	2.4440 (3)	C8—H8C	0.9800
Pd1—Se1	2.4458 (3)	C9—C12	1.514 (4)
Se1—P1	2.1543 (6)	C9—C10	1.525 (4)
Se2—P2	2.1654 (6)	C9—C11	1.527 (3)
P1—N3	1.6132 (18)	C10—H10A	0.9800
P1—N1	1.6773 (19)	C10—H10B	0.9800
P1—N2	1.6855 (18)	C10—H10C	0.9800
P2—N4	1.6093 (19)	C11—H11A	0.9800
P2—N1	1.6799 (18)	C11—H11B	0.9800
P2—N2	1.6856 (19)	C11—H11C	0.9800
N1—C1	1.502 (3)	C12—H12A	0.9800
N2—C5	1.500 (3)	C12—H12B	0.9800
N3—C9	1.508 (3)	C12—H12C	0.9800
N3—H3	0.9099	C13—C16	1.411 (6)
N4—C13	1.499 (3)	C13—C14A	1.437 (7)
N4—H4	0.9099	C13—C15A	1.492 (7)
C1—C2	1.519 (4)	C13—C14	1.522 (6)
C1—C4	1.520 (4)	C13—C15	1.602 (6)
C1—C3	1.523 (4)	C13—C16A	1.607 (7)
C2—H2A	0.9800	C14—H14A	0.9800
C2—H2B	0.9800	C14—H14B	0.9800
C2—H2C	0.9800	C14—H14C	0.9800
C3—H3A	0.9800	C15—H15A	0.9800
C3—H3B	0.9800	C15—H15B	0.9800
C3—H3C	0.9800	C15—H15C	0.9800
C4—H4A	0.9800	C16—H16A	0.9800
C4—H4B	0.9800	C16—H16B	0.9800
C4—H4C	0.9800	C16—H16C	0.9800
C5—C7	1.521 (4)	C14A—H14D	0.9800
C5—C6	1.522 (3)	C14A—H14E	0.9800
C5—C8	1.523 (3)	C14A—H14F	0.9800
C6—H6A	0.9800	C15A—H15D	0.9800
C6—H6B	0.9800	C15A—H15E	0.9800
C6—H6C	0.9800	C15A—H15F	0.9800
C7—H7A	0.9800	C16A—H16D	0.9800
C7—H7B	0.9800	C16A—H16E	0.9800
C7—H7C	0.9800	C16A—H16F	0.9800
			
Cl2—Pd1—Cl1	91.19 (2)	H8A—C8—H8B	109.5
Cl2—Pd1—Se2	79.364 (18)	C5—C8—H8C	109.5
Cl1—Pd1—Se2	169.114 (17)	H8A—C8—H8C	109.5
Cl2—Pd1—Se1	169.511 (19)	H8B—C8—H8C	109.5
Cl1—Pd1—Se1	79.267 (17)	N3—C9—C12	111.2 (2)
Se2—Pd1—Se1	110.536 (10)	N3—C9—C10	110.8 (2)
P1—Se1—Pd1	103.275 (17)	C12—C9—C10	110.6 (2)
P2—Se2—Pd1	103.496 (18)	N3—C9—C11	104.19 (19)
N3—P1—N1	112.67 (10)	C12—C9—C11	109.9 (2)
N3—P1—N2	114.88 (9)	C10—C9—C11	110.1 (2)
N1—P1—N2	83.97 (9)	C9—C10—H10A	109.5
N3—P1—Se1	114.32 (7)	C9—C10—H10B	109.5
N1—P1—Se1	115.25 (7)	H10A—C10—H10B	109.5
N2—P1—Se1	112.32 (7)	C9—C10—H10C	109.5
N4—P2—N1	113.00 (10)	H10A—C10—H10C	109.5
N4—P2—N2	111.62 (10)	H10B—C10—H10C	109.5
N1—P2—N2	83.89 (9)	C9—C11—H11A	109.5
N4—P2—Se2	117.13 (7)	C9—C11—H11B	109.5
N1—P2—Se2	112.12 (7)	H11A—C11—H11B	109.5
N2—P2—Se2	114.56 (7)	C9—C11—H11C	109.5
C1—N1—P1	131.96 (15)	H11A—C11—H11C	109.5
C1—N1—P2	131.96 (15)	H11B—C11—H11C	109.5
P1—N1—P2	93.88 (9)	C9—C12—H12A	109.5
C5—N2—P1	131.49 (14)	C9—C12—H12B	109.5
C5—N2—P2	131.07 (14)	H12A—C12—H12B	109.5
P1—N2—P2	93.38 (9)	C9—C12—H12C	109.5
C9—N3—P1	134.04 (15)	H12A—C12—H12C	109.5
C9—N3—H3	115.7	H12B—C12—H12C	109.5
P1—N3—H3	110.3	C14A—C13—C15A	117.7 (5)
C13—N4—P2	137.71 (17)	C16—C13—N4	116.3 (3)
C13—N4—H4	112.1	C14A—C13—N4	110.1 (3)
P2—N4—H4	110.2	C15A—C13—N4	110.5 (3)
N1—C1—C2	110.0 (2)	C16—C13—C14	116.9 (4)
N1—C1—C4	108.4 (2)	N4—C13—C14	105.1 (3)
C2—C1—C4	109.7 (2)	C16—C13—C15	110.2 (4)
N1—C1—C3	107.8 (2)	N4—C13—C15	105.9 (3)
C2—C1—C3	111.3 (2)	C14—C13—C15	100.8 (4)
C4—C1—C3	109.5 (2)	C14A—C13—C16A	107.5 (5)
C1—C2—H2A	109.5	C15A—C13—C16A	102.7 (4)
C1—C2—H2B	109.5	N4—C13—C16A	107.5 (3)
H2A—C2—H2B	109.5	C13—C14—H14A	109.5
C1—C2—H2C	109.5	C13—C14—H14B	109.5
H2A—C2—H2C	109.5	H14A—C14—H14B	109.5
H2B—C2—H2C	109.5	C13—C14—H14C	109.5
C1—C3—H3A	109.5	H14A—C14—H14C	109.5
C1—C3—H3B	109.5	H14B—C14—H14C	109.5
H3A—C3—H3B	109.5	C13—C15—H15A	109.5
C1—C3—H3C	109.5	C13—C15—H15B	109.5
H3A—C3—H3C	109.5	H15A—C15—H15B	109.5
H3B—C3—H3C	109.5	C13—C15—H15C	109.5
C1—C4—H4A	109.5	H15A—C15—H15C	109.5
C1—C4—H4B	109.5	H15B—C15—H15C	109.5
H4A—C4—H4B	109.5	C13—C16—H16A	109.5
C1—C4—H4C	109.5	C13—C16—H16B	109.5
H4A—C4—H4C	109.5	H16A—C16—H16B	109.5
H4B—C4—H4C	109.5	C13—C16—H16C	109.5
N2—C5—C7	112.62 (19)	H16A—C16—H16C	109.5
N2—C5—C6	107.7 (2)	H16B—C16—H16C	109.5
C7—C5—C6	110.1 (2)	C13—C14A—H14D	109.5
N2—C5—C8	106.93 (18)	C13—C14A—H14E	109.5
C7—C5—C8	110.7 (2)	H14D—C14A—H14E	109.5
C6—C5—C8	108.7 (2)	C13—C14A—H14F	109.5
C5—C6—H6A	109.5	H14D—C14A—H14F	109.5
C5—C6—H6B	109.5	H14E—C14A—H14F	109.5
H6A—C6—H6B	109.5	C13—C15A—H15D	109.5
C5—C6—H6C	109.5	C13—C15A—H15E	109.5
H6A—C6—H6C	109.5	H15D—C15A—H15E	109.5
H6B—C6—H6C	109.5	C13—C15A—H15F	109.5
C5—C7—H7A	109.5	H15D—C15A—H15F	109.5
C5—C7—H7B	109.5	H15E—C15A—H15F	109.5
H7A—C7—H7B	109.5	C13—C16A—H16D	109.5
C5—C7—H7C	109.5	C13—C16A—H16E	109.5
H7A—C7—H7C	109.5	H16D—C16A—H16E	109.5
H7B—C7—H7C	109.5	C13—C16A—H16F	109.5
C5—C8—H8A	109.5	H16D—C16A—H16F	109.5
C5—C8—H8B	109.5	H16E—C16A—H16F	109.5
			
N3—P1—N1—C1	64.6 (2)	Se1—P1—N3—C9	9.1 (3)
N2—P1—N1—C1	179.1 (2)	N1—P2—N4—C13	131.3 (3)
Se1—P1—N1—C1	−69.0 (2)	N2—P2—N4—C13	−136.2 (3)
N3—P1—N1—P2	−131.16 (9)	Se2—P2—N4—C13	−1.3 (3)
N2—P1—N1—P2	−16.68 (9)	P1—N1—C1—C2	51.1 (3)
Se1—P1—N1—P2	95.18 (7)	P2—N1—C1—C2	−107.5 (3)
N4—P2—N1—C1	−68.2 (2)	P1—N1—C1—C4	171.10 (18)
N2—P2—N1—C1	−179.1 (2)	P2—N1—C1—C4	12.5 (3)
Se2—P2—N1—C1	66.8 (2)	P1—N1—C1—C3	−70.4 (3)
N4—P2—N1—P1	127.60 (10)	P2—N1—C1—C3	131.0 (2)
N2—P2—N1—P1	16.68 (9)	P1—N2—C5—C7	−74.2 (3)
Se2—P2—N1—P1	−97.38 (7)	P2—N2—C5—C7	76.9 (3)
N3—P1—N2—C5	−72.6 (2)	P1—N2—C5—C6	164.2 (2)
N1—P1—N2—C5	175.2 (2)	P2—N2—C5—C6	−44.7 (3)
Se1—P1—N2—C5	60.3 (2)	P1—N2—C5—C8	47.6 (3)
N3—P1—N2—P2	128.84 (10)	P2—N2—C5—C8	−161.39 (17)
N1—P1—N2—P2	16.61 (9)	P1—N3—C9—C12	−63.8 (3)
Se1—P1—N2—P2	−98.24 (7)	P1—N3—C9—C10	59.6 (3)
N4—P2—N2—C5	72.4 (2)	P1—N3—C9—C11	177.9 (2)
N1—P2—N2—C5	−175.3 (2)	P2—N4—C13—C16	−8.1 (5)
Se2—P2—N2—C5	−63.7 (2)	P2—N4—C13—C14A	−179.4 (4)
N4—P2—N2—P1	−128.94 (10)	P2—N4—C13—C15A	48.9 (5)
N1—P2—N2—P1	−16.59 (9)	P2—N4—C13—C14	−139.3 (4)
Se2—P2—N2—P1	94.97 (7)	P2—N4—C13—C15	114.6 (4)
N1—P1—N3—C9	−125.0 (2)	P2—N4—C13—C16A	−62.5 (4)
N2—P1—N3—C9	141.1 (2)		

### Hydrogen-bond geometry (Å, º)

**Table:**

*D*—H···*A*	*D*—H	H···*A*	*D*···*A*	*D*—H···*A*
N4—H4···Cl1^i^	0.91	2.45	3.317 (2)	159
N3—H3···Cl1^i^	0.91	2.57	3.4160 (19)	155
C16—H16*A*···Cl2^ii^	0.98	2.82	3.746 (5)	157
C14*A*—H14*E*···Cl2^ii^	0.98	2.82	3.742 (7)	157

Symmetry codes: (i) *x*−1/2, *y*, −*z*+1/2; (ii) *x*−1/2, −*y*+1/2, −*z*+1.
